# Process Temperature Control for Low Dishing in CMP

**DOI:** 10.3390/ma18194461

**Published:** 2025-09-24

**Authors:** Yeongil Shin, Jongmin Jeong, Jiho Shin, Haedo Jeong

**Affiliations:** Graduated School of Mechanical Engineering, Pusan National University, Busan 46703, Republic of Korea; oil5108@pusan.ac.kr (Y.S.);

**Keywords:** chemical mechanical planarization (CMP), dishing, temperature, selectivity, vortex tube, pad cooling system

## Abstract

Growing demand for high-performance system semiconductors has highlighted the importance of hybrid bonding, where precise control of copper dishing is essential. This requirement reinforces the role of chemical mechanical planarization (CMP). Many studies have sought to control dishing by modifying slurry chemistry or adjusting mechanical parameters, but these approaches have not been sufficient. This study addresses the overlooked effect of process temperature and demonstrates its role in integrating both chemical and mechanical behaviors in CMP. Removal rates of Cu, Ta, and SiO_2_ films were evaluated through blanket wafer experiments, and all exhibited Arrhenius-type behavior as a function of temperature and activation energy. The results showed that maintaining the process temperature at 30 °C balanced selectivity and minimized dishing on patterned wafers. To enable precise temperature control, a vortex-tube-based pad cooling system was developed. Without temperature control, dishing increased by 12 nm in the 100 µm pattern and 16 nm in the 50 µm pattern. With temperature control, dishing was reduced to 4 nm and below 1 nm, respectively. These results demonstrate that process temperature is a key parameter for controlling selectivity and ensuring precise dishing control, which is critical to meeting the requirements of hybrid bonding.

## 1. Introduction

Rapid growth of artificial intelligence, high-performance computing, and big data analytics has further increased the demand for advanced system semiconductors with superior performance and power efficiency [1]. As conventional two-dimensional chip architectures approach their limits, three-dimensional (3D) integration has gained considerable attention [2]. In particular, heterogeneous integration based on chiplet architectures has highlighted the importance of hybrid bonding. This bonding method, which simultaneously connects metals and dielectrics, provides clear advantages in electrical performance and energy efficiency and is being actively introduced into next-generation high bandwidth memory (HBM). For reliable implementation, stringent control of surface topography, especially copper dishing, has become increasingly important [3]. This trend further reinforces the role of chemical mechanical planarization (CMP) in achieving the required surface quality.

Many studies have attempted to mitigate copper dishing during CMP through modification of slurry components or optimization of mechanical parameters. Wang et al. [4] adjusted slurry composition and pH conditions to improve selectivity, achieving about 15% reduction in dishing. Zeng et al. [5] proposed a non-Prestonian slurry by adjusting pH and inhibitors, explaining the pressure-dependent removal of passivation films, but dishing still increased under overpolishing conditions. Shin et al. [6] investigated the role of slurry additives and temperature, showing improvements in dishing at elevated temperatures. Wu and Yan [7] analyzed the effect of process variables such as selectivity, pressure, and pad modulus, and reported that pads with a high elastic modulus (≈150 MPa) could suppress dishing. Ji and Chui [8] studied pattern-dependent effects on dishing behavior and proposed design guidelines from a layout perspective. Park et al. [9] developed a structured-surface polishing pad, demonstrating up to 69% reduction in dishing compared with commercial pads. Despite these diverse approaches, dishing could not be sufficiently suppressed. These limitations suggest that another factor may govern dishing behavior. In particular, the role of process temperature has not been fully addressed, despite its significant influence on removal characteristics.

Several studies have investigated the effect of temperature on CMP processes. Mudhivarthi et al. [10] reported that higher temperature softens the pad and reduces slurry viscosity, which increases the contact area, friction, and shear force, leading to a higher removal rate. Kim et al. [11] further showed that elevating pad conditioning temperature influences slurry chemistry, particle dispersion, pad surface structure, and mechanical properties. In particular, the storage modulus of a polyurethane pad decreased from about 35 MPa at 20 °C to about 10 MPa at 90 °C, with a nearly linear reduction up to 70 °C followed by saturation. Khanna et al. [12] provided a fundamental understanding of the impact of pad thermal instability, demonstrating that a larger ratio between low and high temperature storage modulus produced sharper increases in removal rate with temperature, whereas a smaller ratio yielded more stable behavior. Mu et al. [13] showed that temperature not only increases the removal rate but also shifts the dominant removal mechanism. By analyzing soft-pad polishing, they proposed the ratio of chemical to mechanical rate constants as an indicator of balance between removal pathways, and demonstrated that higher temperatures drive a transition from chemically dominated to mechanically dominated behavior. Ilie et al. [14] emphasized that CMP is a process where mechanical abrasion and chemical reactions are strongly coupled through temperature: friction raises the temperature, the elevated temperature accelerates chemical reaction rates, and the combined effect governs the overall removal rate.

Therefore, temperature can be regarded as an integrated parameter that represents both the chemical and mechanical behavior of CMP. Based on this perspective, the present study simplified the process by fixing slurry composition and mechanical conditions, while treating process temperature as the key parameter for controlling selectivity. Differences in activation energy among copper, barrier metals, and oxides were used to define the relationship between temperature and selectivity. A dedicated temperature control system was developed for precise temperature adjustment, and its effectiveness was validated through patterned wafer polishing and dishing analysis.

## 2. Materials and Methods

### 2.1. Experimental Setup and Polishing Conditions

**Figure 1:**
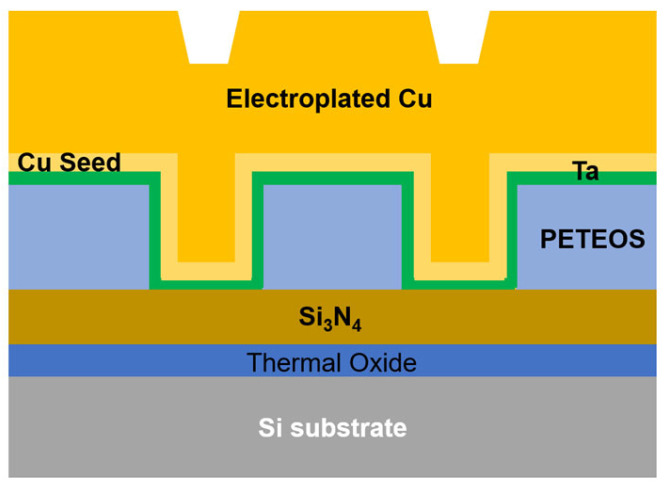
Cross-sectional structure of the patterned wafer used for CMP experiments.

Figure 1 shows the cross-sectional view of a 200 mm wafer (SKW 6-3.18C) used in this study [15]. Patterned wafers were used to verify the effectiveness of dishing control with the pad cooling system. During the barrier removal step, the CMP target materials were Ta, SiO_2_, and Cu. To evaluate the removal rate of each material as a function of activation energy and temperature, preliminary experiments were performed using 200 mm blanket wafers. CMP experiments were conducted with a POLI-500 equipment from G&P (Busan, Republic of Korea), and the pad temperature was monitored using a mounted infrared sensor. Detailed experimental conditions are summarized in Table 1.

The thicknesses of the removed Cu and Ta films were determined by comparing surface resistivity before and after CMP using the CMT-SR5000 four-point probe system (AIT, Suwon, Republic of Korea). The thickness of the SiO_2_ film was measured using the ST5030-SL reflectometer (K-MAC, Daejeon, Republic of Korea). For blanket wafers, thickness was measured at 41 equally spaced points across the wafer, excluding the outer 5 mm edge, and the average value was calculated. The dishing depth of the patterned wafers was measured with an Alpha-Step D-600 stylus profiler (KLA, Milpitas, CA, USA).

**Figure 2:**
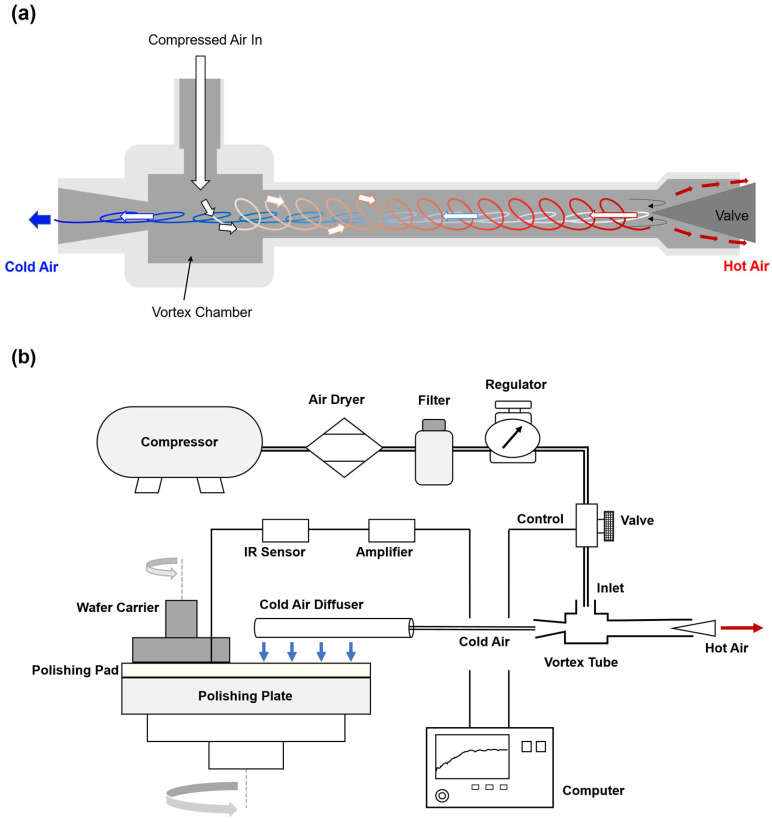
Schematic diagram of the vortex-tube-based cooling system: (**a**) Structure and operating principle of the vortex tube; (**b**) Configuration of the pad surface cooling system with diffuser and control unit.

Process temperature was controlled using a vortex tube. The vortex tube, as shown in Figure 2a, is a simple device with no moving parts that separates compressed air into cold and hot streams, and it has been widely applied in various industries [16,17]. In this study, an air diffuser was fabricated and mounted to distribute the cold air uniformly along the radial direction of the pad, and the overall cooling system was designed as shown in Figure 2b. Compressed air at 4.5 bar was supplied to the vortex tube. The outlet temperature of the diffuser dropped to approximately 5 °C within 3 s and gradually increased with distance from the outlet. A control valve was installed at the tube inlet to maintain the set process temperature.

### 2.2. Control of Process Temperature

Prior studies have reported different evaluations of heat generation in CMP. White et al. [18] calculated the energy flow rate and found that frictional heating was approximately 200–300 W, whereas chemical heating was only about 1 W, indicating that friction is the dominant contributor. However, their experiments were performed under neutral slurry conditions without oxidizers, even though oxidizers play a crucial role in metal CMP. In contrast, Wang et al. [19] observed that pad temperature rise varied strongly with slurry chemistry, such as oxidizer concentration and pH, suggesting that exothermic reactions can substantially accelerate heating.

**Figure 3:**
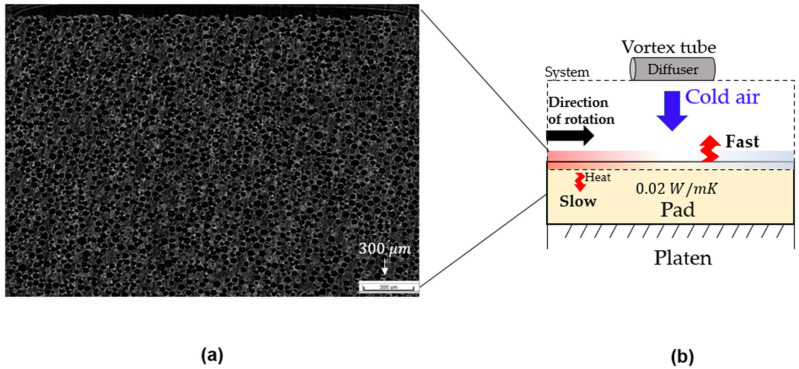
(**a**) Cross-sectional micro-CT image of the porous polyurethane pad. (**b**) Schematic of process temperature control using a vortex-tube cooling system, illustrating the low thermal conductivity of the pad (≈0.02 W/m·K) and surface heat removal by forced cold airflow.

In this study, the slurry used was not designed for high selectivity, and thus the frictional heating within the operating window was estimated to be approximately 200–300 W, while chemical heat release was assumed to be negligible. This energy is transferred into the pad surface and the slurry film. The porous polyurethane pad, shown in Figure 3a, has a very low thermal conductivity (≈0.02 W/m·K), so most of the generated heat remains near the surface rather than being conducted into the platen. The platen has a diameter of 500 mm and rotates at 93 rpm. Under these conditions, the same pad surface region re-contacts the wafer in less than a second, leaving little opportunity for natural convection to dissipate heat during rotation. In our experiments, the temperature difference under these conditions was within tenths of a degree.

The vortex-tube cooling system employed in this study provides a nominal cooling capacity ranging from 180 to 730 W, depending on supply pressure and diffuser standoff distance. Because direct measurement of effective cooling capacity under all conditions is challenging, the system was empirically tuned to optimal settings for the present experiments. Given the broad range of effective cooling capacity, the vortex tube can be adapted for various operating conditions. Consequently, the accumulated surface heat was effectively removed by forced convection from the vortex-tube air stream, enabling stable process temperature control during polishing, as shown in Figure 3b.

## 3. Results and Discussion

### 3.1. Integrated Role of Temperature in CMP

Process temperature is a key parameter that governs both chemical and mechanical behaviors in CMP. Although it is difficult to define thermal variations during CMP precisely, it is evident that factors such as contact interface, material properties, mechanical conditions, chemical composition, and lubrication characteristics are involved. Therefore, polishing temperature is not merely a result variable but provides important insights into polishing interactions, ultimately leading to changes in removal rate. For this reason, the present study considers temperature as an integrated parameter for explaining the complex mechanisms of CMP.

Numerous studies have demonstrated that temperature serves as a key parameter linking the chemical and mechanical aspects of CMP. DeNardis et al. [20,21] showed that the oxidation rate of copper follows a temperature-dependent behavior consistent with Arrhenius kinetics, indicating that higher temperatures accelerate surface reactions. Non-oxidized soft copper exhibits almost no material removal during CMP [22,23], which supports the view that the reaction rate of a material directly corresponds to its removal rate. Oh and Seok [24] further emphasized that frictional heating modifies pad properties and contact mechanics while simultaneously increasing the rate of chemical reactions, thereby coupling mechanical abrasion and chemical removal through process temperature. Other modeling studies have also highlighted that thermal effects must be considered to properly describe material removal behavior in CMP [25,26].

In this study, the analysis was simplified by focusing exclusively on the relationship among process temperature, activation energy, and removal rate. This approach is justified by prior research showing that temperature mediates the coupling of chemical and mechanical effects, and by the use of the Arrhenius equation as an effective tool to describe this dependence.

During CMP, the slurry chemically reacts with the wafer film material and facilitates surface removal. The reaction rate is a function of temperature, and the activation energy of each material determines its sensitivity to thermal variations. Materials with higher activation energies respond more strongly to temperature changes. In general, chemical reaction rates are described by the Arrhenius equation. However, since reaction rates cannot be measured directly in CMP, this study employed removal rate as a surrogate variable for reaction rate, applying the Arrhenius relation to CMP as expressed in Equation (1).

(1) $$RR=Ae^{\left(-\frac{E_{a}}{RT}\right)}$$

**Figure 4:**
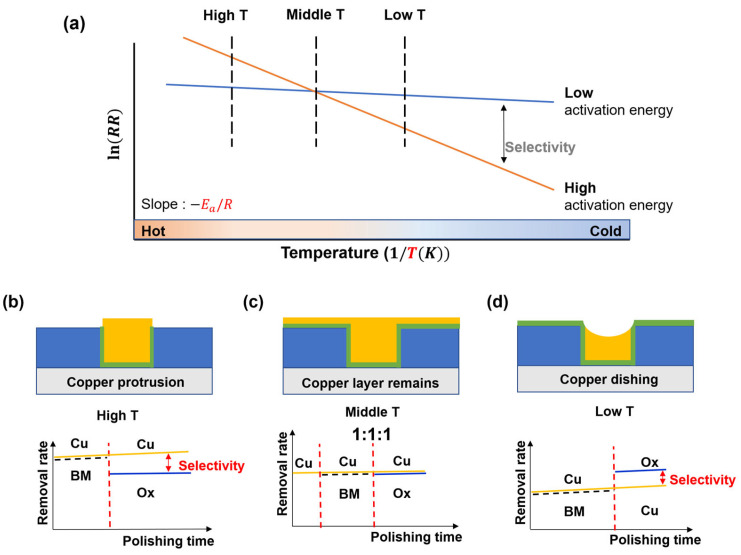
Conceptual schematic of process temperature control in CMP: (**a**) Arrhenius-type temperature dependence of removal rate; (**b**) Copper protrusion removal at high temperature; (**c**) Planar surface maintained at intermediate temperature; (**d**) Copper dishing compensation at low temperature.

Here, *RR* denotes the removal rate (Å/min), $E_{a}$ the activation energy (J/mol), *R* the gas constant (8.314 J/K·mol), *T* the average process temperature (K), and *A* the frequency factor constant (Å/min). Based on this equation, the conceptual approach of this study is illustrated in Figure 4. Figure 4a shows the Arrhenius-type schematic, where differences in activation energy among materials lead to distinct temperature-dependent removal behaviors. In Figure 4b–d, the materials are denoted as Cu (copper), BM (barrier metal), and Ox (SiO_2_) for clarity. When copper protrusion is present, polishing should be performed at high temperatures, where copper selectivity is high, as shown in Figure 4b. To maintain a flat surface, polishing must be conducted at intermediate temperatures, where the selectivity is balanced, as illustrated in Figure 4c. When severe dishing is present, polishing should be carried out at lower temperatures, where the reduced copper selectivity enables compensation of the recessed surface, as shown in Figure 4d.

### 3.2. Temperature-Removal Rate Relationship

**Figure 5:**
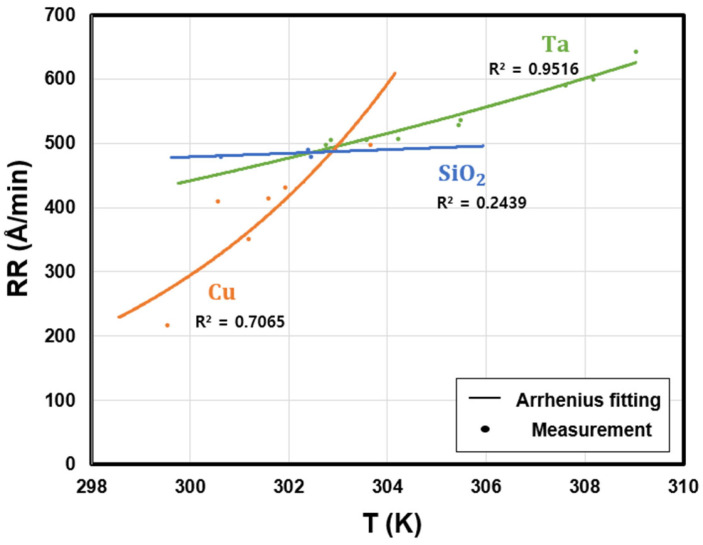
Removal rates of Cu, Ta, and SiO_2_ as a function of temperature.

In order to investigate the relationship between the removal rate and activation energy of Cu, Ta, and SiO_2_ films, CMP experiments were performed using blanket wafers. Temperature control was achieved through a vortex tube cooling system, and the process temperature was measured on the pad surface immediately after contact with the wafer using an infrared sensor. Figure 5 shows the variation in removal rates of the three materials as a function of the average process temperature. For SiO_2_, the low coefficient of determination (R^2^) can be attributed to its low activation energy, which allows sufficient chemical reaction to form a hydrated layer under typical CMP process temperatures. As a result, SiO_2_ does not exhibit strong temperature sensitivity. Within the temperature range accessible in the present experiments, the removal rate differed by only a few Å/min, indicating negligible variation with temperature. Rather, SiO_2_ provides a baseline in the slurry system and serves as an indicator to confirm whether the temperature-dependent removal behavior of metallic films falls within the controllable temperature range in industrial CMP. In this case, the curve of metal films removal rate are primarily a function of the H_2_O_2_ concentration in the slurry, whereas the oxide removal rate remains essentially unaffected by oxidizer.

**Figure 6:**
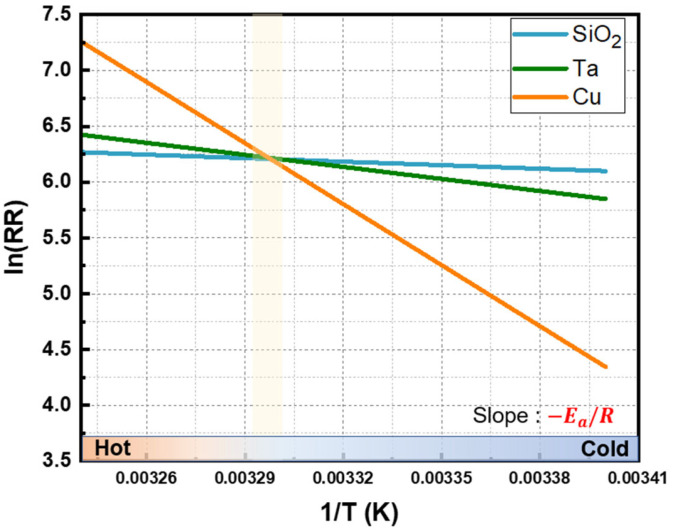
Arrhenius plot of Cu, Ta, and SiO_2_ as a function of temperature.

As a result of the experiments, the removal rates of all three materials increased exponentially with rising temperature, which was consistent with the Arrhenius equation. By taking the natural logarithm of both sides, the relationship can be expressed as a linear form, as shown in Equation (2), and the activation energy of each material can be determined from the slope, as shown in Figure 6.

(2) $$\ln RR=-\frac{E_{a}}{R}\cdot \frac{1}{T}+\ln A$$

The calculated activation energies were 8.75 kJ/mol for SiO_2_, 29.9 kJ/mol for Ta, and 151.7 kJ/mol for Cu, while the corresponding ln*A* values were 9.7, 18.1, and 66.3, respectively. Cu exhibited the highest activation energy, indicating that a large energy barrier must be overcome before it can react with the slurry to form oxides. In contrast, SiO_2_ showed a very low activation energy, implying that the reaction can proceed even without elevated temperatures, as the reactant molecules can readily acquire sufficient energy. Ta exhibited an intermediate behavior and followed the typical trend observed for metallic materials.

The slurry used in this study exhibited removal rates of approximately 400–500 Å/min for all three materials under the conditions listed in Table 1, but their temperature-dependent behaviors showed clear differences. The Arrhenius plots of the three materials intersected at around 30 °C, where the removal rates converged to approximately 500 Å/min, resulting in a nearly 1:1:1 selectivity ratio. Maintaining this temperature ensured a balanced removal rate among the materials and was found to be effective in minimizing dishing during the CMP process.

Although the commercial barrier slurry is formulated to provide nearly identical selectivity, the actual process temperature rises during polishing, which significantly alters the temperature-dependent removal rates. Therefore, without controlling the process temperature, it is not possible to achieve very low dishing.

### 3.3. Temperature Control for Low Dishing

Cu CMP is generally divided into a bulk removal step and a barrier removal step. In this study, the effect of process temperature on copper pattern dishing was investigated during barrier CMP. For this purpose, wafers with the barrier layer exposed were prepared by removing the bulk copper layer in advance. The results obtained under uncontrolled process temperature were compared with those under a controlled temperature condition of approximately 30 °C. This temperature was identified from the blanket wafer experiments as the point where selectivity among the materials was balanced.

**Figure 7:**
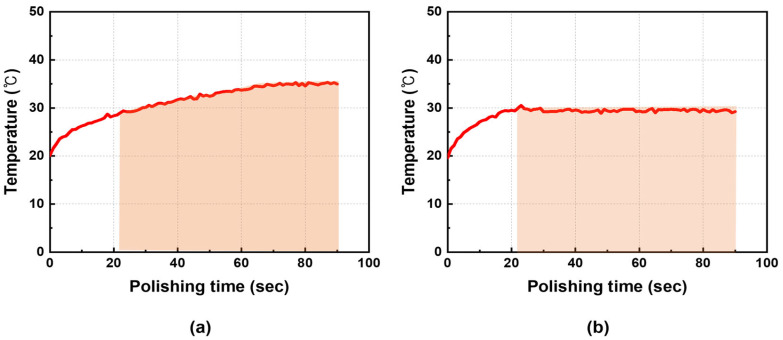
Process temperature profiles during CMP: (**a**) Without cooling, the temperature gradually increased to above ~36 °C; (**b**) With cooling, the temperature was stabilized and maintained at 30 °C throughout polishing.

CMP was performed for 90 s, and the process temperature was measured on the pad surface immediately after contact with the wafer using an IR monitoring sensor. The measurement point was located at the mid-radius position of the pad. Without the cooling system, the temperature continuously increased with polishing time, as shown in Figure 7a. In contrast, Figure 7b demonstrates that the cooling system effectively maintained the target temperature. The average process temperature was maintained at approximately 30 °C under the controlled condition, whereas in the uncontrolled condition it was more than 3 °C higher, with the difference increasing as polishing progressed. This trend is expected to accelerate dishing.

**Figure 8:**
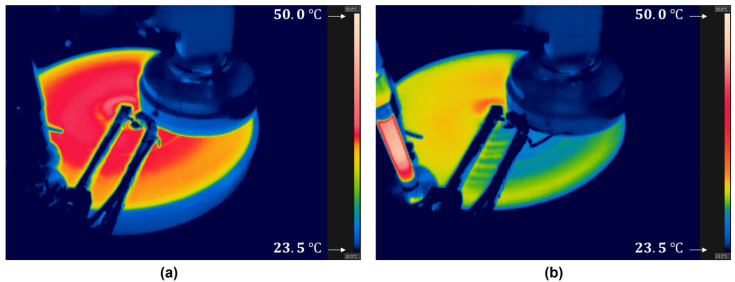
Infrared images of pad surface temperature distribution during CMP with wafer contact: (**a**) Without temperature control; (**b**) With temperature control. The entire pad surface was captured to enable a direct comparison of the temperature distribution with and without the cooling system.

Figure 8 shows infrared images of the pad surface temperature distribution during CMP under wafer contact. The thermal camera captured the entire pad surface, allowing a direct comparison of temperature distribution between the condition Figure 8a without cooling and Figure 8b with cooling. These images clearly demonstrate the effectiveness of the vortex-tube-based cooling system. Without the cooling system, the average pad surface temperature immediately after wafer contact differed by only about 0.5 °C from that before contact, as shown in Figure 8a. In contrast, when the cooling system was applied, the pad surface temperature decreased rapidly from approximately 30 °C to 27.5 °C after passing through a single diffuser and further to 26.5 °C after passing through a second diffuser, as shown in Figure 8b. The slight non-uniform temperature distribution observed after cooling in Figure 8b appears to result from the cooling outlets, which had a diameter of 4 mm and were arranged at 20 mm intervals. Nevertheless, the pad surface temperature after wafer contact was found to be uniform. To further improve uniformity, the outlets can be arranged with higher density or milled into a continuous line-shaped slot along one side of the diffuser.

**Figure 9:**
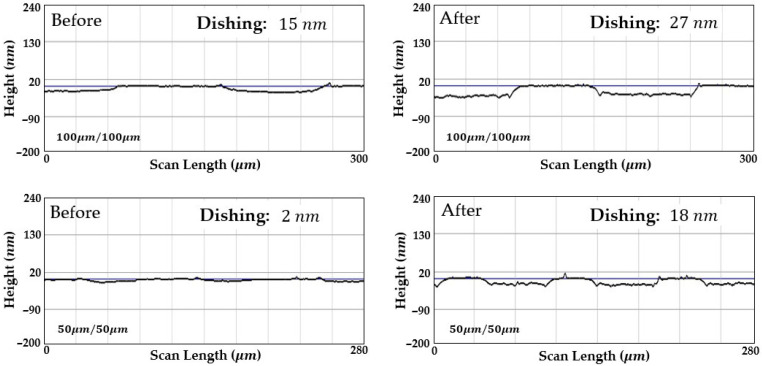
Dishing profiles of patterned wafers before and after CMP without temperature control. As the process temperature entered the hot zone, the selectivity shifted toward high Cu, leading to an increase in dishing by more than 10 nm.

**Figure 10:**
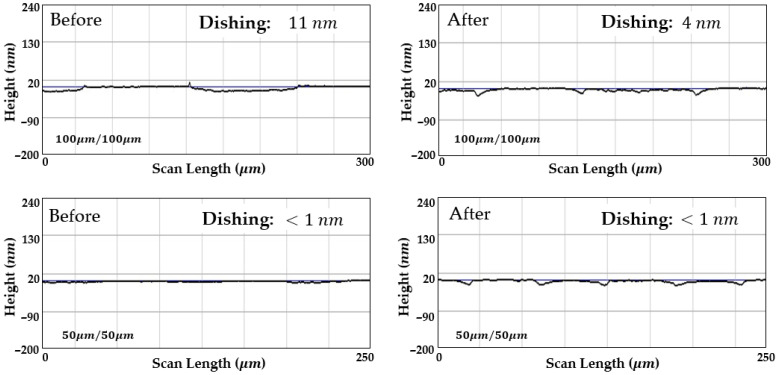
Dishing profiles of patterned wafers before and after CMP with temperature control. Under controlled temperature conditions, both patterns remained within a low dishing range.

Dishing was evaluated by measuring the wafer surface profile before and after CMP using a stylus profiler. The measurement point was located approximately 50 mm from the wafer center, and dishing was defined as the height difference between regions of different materials. Figure 9 and Figure 10 compare surface profiles before and after CMP under uncontrolled temperature and controlled temperature conditions, respectively. The evaluation was carried out on patterns with a line density of 50% and line widths of 100 μm and 50 μm. The blue line denotes the baseline: Ta layer before polishing and SiO_2_ layer after polishing. The Cu regions appear lower than or close to this reference in both cases.

Without temperature control, the process temperature rapidly exceeded 30 °C, placing CMP in the hot zone shown in Figure 6. In this region, the selectivity of Cu increased, which in turn intensified dishing. After 90 s of polishing, the dishing depth increased from 15 nm to 27 nm in the 100 μm pattern and from 2 nm to 18 nm in the 50 μm pattern.

In contrast, with temperature control, the process temperature was maintained at approximately 30 °C, and the selectivity remained constant throughout CMP. Under this condition, the dishing depth in the 100 μm pattern decreased from 11 nm to 4 nm. At the temperature giving balanced selectivity, Ta and SiO_2_ regions experienced higher local pressure during planarization. As a result, their removal rate exceeded that of Cu, which reduced dishing. In the 50 μm pattern, dishing remained below 1 nm both before and after CMP.

In Figure 9, contrary to expectations, the increase in dishing was about 4 nm larger in the smaller pattern than in the larger one. This discrepancy cannot be explained by frictional considerations alone. While the limitations of stylus profilometry at the nanometer scale are one possible cause, additional effects such as local pressure distribution, edge effects, and pad deformation may also contribute. At present, the analysis has focused on the material characteristics, but to achieve a more accurate approach, we are developing mathematical analyses and predictive modeling.

**Figure 11:**
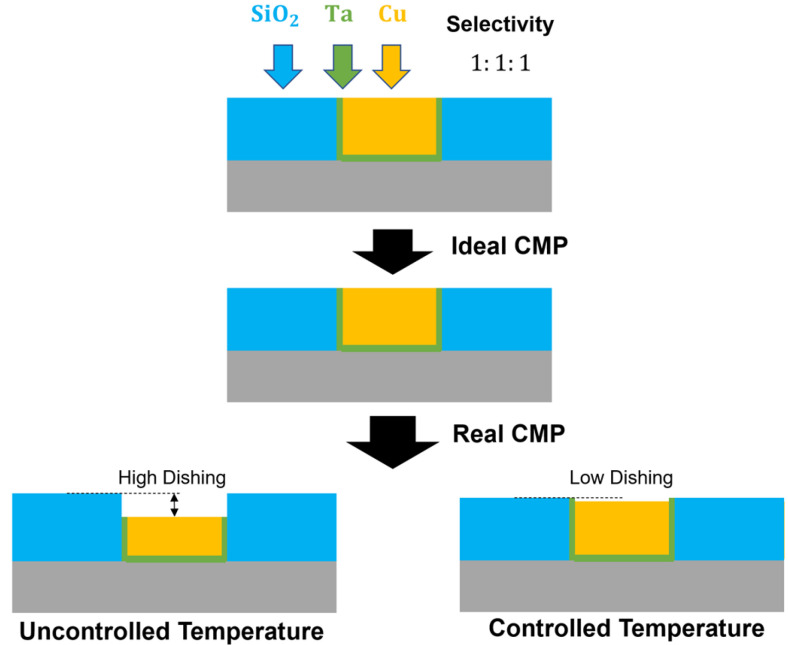
Comparison of dishing outcomes: ideal CMP at balanced selectivity versus real CMP under uncontrolled and controlled temperature conditions.

Nevertheless, these dishing results are consistent with the relationship between temperature and selectivity derived from the blanket wafer experiments. As shown in Figure 6, copper exhibits much greater sensitivity to temperature variations than other materials, showing large differences in removal rate even with minor changes in process temperature. In practice, CMP temperature increases nonlinearly with polishing time, and the rate of this increase becomes faster with larger wafer diameters, higher applied pressures, and faster chemical reactions. Such nonlinear behavior explains why earlier studies that did not account for process temperature failed to achieve precise dishing control, as illustrated in Figure 11. Therefore, process temperature control was demonstrated to be effective in fulfilling the requirements for precise dishing control.

In practice, very low dishing is often not achieved with a commercial slurry that is designed to provide near-equal selectivity, because under uncontrolled conditions the process drifts to a hotter zone where copper shows much stronger temperature sensitivity than Ta and SiO_2_. Maintaining the process temperature at the balance point (~30 °C under the present conditions) stabilizes the selectivity and prevents drift toward copper-high removal, thereby keeping dishing within 1–4 nm.

## 4. Conclusions

This study demonstrated that process temperature plays an essential role in explaining the complex behaviors of CMP. The removal rates of Cu, Ta, and SiO_2_ films followed the Arrhenius relation, and differences in activation energy quantitatively revealed the sensitivity of each material to temperature variations. Under the experimental conditions, the process temperature of 30 °C was identified as the balance point where the selectivity among the three materials converged, enabling uniform removal rates through precise temperature control.

In patterned wafers, temperature control effectively suppressed dishing, maintaining dishing amount as low as 4 nm in the 100 μm pattern and below 1 nm in the 50 μm pattern. In contrast, uncontrolled processes exhibited accelerated dishing of more than 10 nm compared with the initial state. The vortex tube-based pad cooling system successfully enabled precise temperature adjustment, confirming the feasibility of direct process temperature control in CMP.

These results indicate that process temperature is a key parameter for selectivity control and precise dishing management. By integrating chemical and mechanical influences into a single controllable variable, this approach provides a practical and effective pathway for achieving the stringent surface quality requirements of next-generation semiconductor processes.

## Figures and Tables

**Table 1:** Experimental conditions.

Parameters	Conditions
Polishing Pad	Hybrid Pore PAD (Stacked, KPX)
Slurry	$\mathrm{Barrier}\;\mathrm{slurry}\;(H_{2}O_{2}$: 0.5 wt%, Commercial)
Pressure	Wafer/R-ring 2/3 psi
Velocity	Carrier/Platen: 87/93 rpm
Slurry flow rate	150 mL/min
Oscillation of carrier	Enable
Conditioning	In situ

## Data Availability

The original contributions presented in this study are included in the article. Further inquiries can be directed to the corresponding author.

## Abbreviations

The following abbreviations are used in this manuscript:

CMP	Chemical mechanical planarization
RR	Removal rate
BM	Barrier metal
Ox	SiO_2_
